# Biocompatibility and Microstructure-Based Stress Analyses of TiNbZrTa Composite Films

**DOI:** 10.3390/ma15010029

**Published:** 2021-12-21

**Authors:** Bo-Wei Lai, Yin-Yu Chang, Tzong-Ming Shieh, Heng-Li Huang

**Affiliations:** 1 School of Dentistry, China Medical University, Taichung 404, Taiwan; brian860303@gmail.com; 2 Department of Mechanical and Computer-Aided Engineering, National Formosa University, Yunlin 632, Taiwan; yinyu@nfu.edu.tw; 3 Department of Bioinformatics and Medical Engineering, Asia University, Taichung 413, Taiwan

**Keywords:** TiNbZr, TiNbZrTa, TiNbZrTa(N), TiNbZrTa-O, TiNbZrTa(N)-O coatings, cytotoxicity, cell viability, cell morphology, bone differentiation, RT-qPCR analysis, microstructure stress analysis

## Abstract

Background: the clinical application of orthopedic or dental implants improves the quality of the lives of patients. However, the long-term use of implants may lead to implant loosening and related complications. The purpose of this study is to deposit titanium (Ti)-niobium (Nb)-zirconium (Zr)-tantalum (Ta) alloys on the surface of Ti-6Al-4V to increase structural strength and biocompatibility for the possible future application of implants. Materials and methods: Ti, Nb, Zr, and Ta served as the materials for the surface modification of the titanium alloy. TiNbZr and TiNbZrTa coatings were produced using cathodic arc evaporation, and a small amount of nitrogen was added to produce TiNbZrTa(N) film. Annealing and oxidation were then conducted to produce TiNbZrTa-O and TiNbZrTa(N)-O coatings. In this study, biological tests and finite element analyses of those five alloy films, as well as uncoated Ti-6Al-4V, were performed. Human osteosarcoma cells (MG-63) and mouse fibroblast cells (L-929) were used to analyze cytotoxicity, cell viability, and cell morphology, and the bone differentiation of MG-63 was evaluated in an alkaline phosphatase experiment. Furthermore, for measuring the gene expression level of L-929, reverse transcription quantitative real-time polymerase chain reaction (RT-qPCR) was conducted. The three-dimensional (3D) computational models of the coated and uncoated sample films were constructed using images of transmission electron microscopy and computer-aided design software and, then, the stress distributions of all models were evaluated by finite element analysis. Result: the cytotoxicity test revealed that the surface treatment had no significant cytotoxic effects on MG-63 and L-929 cells. According to the results of the cell viability of L-929, more cell activity was observed in the surface-treated experimental group than in the control group; for MG-63, the cell viability of the coated samples was similar to that of the uncoated samples. In the cell morphology analysis, both MG-63 and L-929 exhibited attached filopodia and lamellipodia, verifying that the cells were well attached. The alkaline phosphatase experiment demonstrated that the surface treatment did not affect the characteristics of early osteogenic differentiation, whereas RT-qPCR analysis showed that surface treatment can promote better performance of L-929 cells in collagen, type I, α1, and fibronectin 1. Finally, the results of the finite element analysis revealed that the coated TiNb interlayer can effectively reduce the stress concentration inside the layered coatings. Conclusions: TiNbZrTa series films deposited using cathodic arc evaporation had excellent biocompatibility with titanium alloys, particularly in regard to soft tissue cells, which exhibited an active performance. The finite element analysis verified that the TiNb interlayer can reduce the stress concentration inside TiNbZrTa series films, increasing their suitability for application in biomedical implants in the future.

## 1. Introduction

Dental implant has been widely accepted as a treatment for replacing missing teeth and restoring human mastication function. In recent years, the use of dental implants has increased considerably, and this restoration option has been continually improved with the introduction of new designs and concepts. However, Avvi-Arber and Zarb [1] noted that abutment screw loosening is the most common mechanical problem, and it is related to component failure, implant inflammation, and potentially serious complications. The rate of abutment screw loosening in the first year following implantation was 5.3%, increasing to 5.8–12.7% after five years of follow-up [2,3]. If this situation is not managed in time, the continual loosening of the abutment screws can result in component breakage.

The development of coatings on metal implants with ideal mechanical properties and biological activity has greatly improved their functionality and applicability in the field of medical implants. Single-metallic coatings have been fashioned to maturity in certain studies. In response to various complex demands, multi-metallic alloy coatings seem to be used to achieve an effect superior to the characteristics of titanium alloys.

Titanium and its alloys have excellent mechanical and anti-corrosion properties as well as good biocompatibility, and they are regarded as the most suitable material for biomedical applications [4,5]. Nonetheless, almost all metallic materials undergo electrochemical corrosion to some extent when they are in contact with corrosive liquids under certain conditions. Therefore, corrosion represents a major challenge with metallic biomaterials. The degradation of substances and the release of ions are the most common results of the reaction between body fluids and implants, causing problems such as inflammation and foreign body cell formation [6], which may lead to implant loosening. Therefore, surface modification can be performed to enhance implant material’s mechanical, biological, and chemical properties.

Since the 1970s, surface coatings have been successfully used to protect materials, particularly in extending the service life of medical parts [7]. However, hard coatings are brittle and often deform under mechanical loading, limiting their application. To meet the requirements for improved coatings such as reducing friction, extending service life, enhancing biological performance, and achieving favorable thermostability in different environments, reinforced and applicable coatings have been developed. Through the application of alloying with appropriate materials, different properties of the coatings can be adjusted to the desired value. Composite materials with structures in the nanometer range, such as multilayer and isotropic nanocomposite coatings, exhibit properties that cannot be obtained through the use of a single coating material [8].

This study used cathodic arc evaporation to deposit titanium (Ti)- niobium (Nb)- zirconium (Zr)- tantalum (Ta)-based alloys with a small amount of nitrogen doping on Ti-6Al-4V plates. Annealing and oxidation were then conducted to modify the surface of the composite coatings. In addition to improving the mechanical properties of the material, this study analyzed whether the composite coatings could provide the biocompatibility of soft and hard tissues, assessed the cell adhesion status and early osteogenic differentiation, and identified what type of gene expression in soft tissues affects cellular performance through reverse transcription quantitative real-time polymerase chain reaction (RT-qPCR). This study also adopted a computer simulation–finite element analysis to explore stress distribution within the structure of different alloy coatings.

## 2. Materials and Methods

### 2.1. Experimental Sample

**Figure 1:**
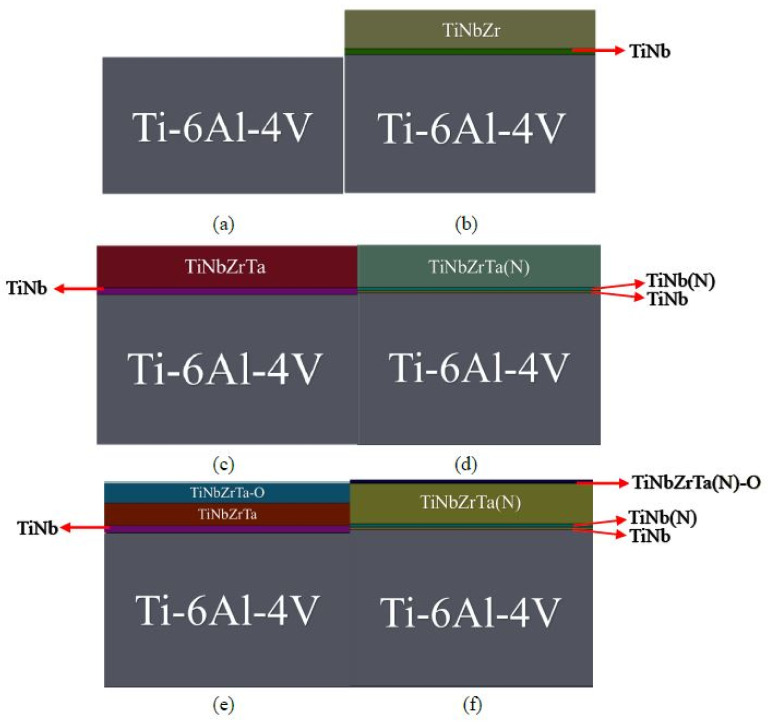
Diagram of the coating structure of (**a**) control Ti-6Al-4V (without coating); (**b**) TiNbZr; (**c**) TiNbZrTa; (**d**) TiNbZrTa(N); (**e**) TiNbZrTa-O; (**f**) TiNbZrTa(N)-O. (**b**–**f**) all the coatings are deposited on the same material, Ti-6Al-4V.

The instrument employed in this experiment was a cathodic arc evaporation system used to conduct thermal evaporation, a method of physical vapor deposition. This system can simultaneously deposit three target materials into alloys, which, in this case, were titanium-niobium alloy (Ti:Nb = 66:34 at%), pure zirconium (Zr), and pure tantalum (Ta). The distance between the target material and the test piece was 170 mm. The coating can achieve a favorable deposition rate and uniformity through the revolution of the rotating stand and rotation of the test piece during coating deposition. The control group in this experiment was Ti-6Al-4V, which was used as the substrate to deposit TiNbZr, TiNbZrTa, and—with a small amount of nitrogen doping, annealing, and oxidation—TiNbZrTa(N), TiNbZrTa-O, and TiNbZrTa(N)-O. The five experimental groups are as depicted in Figure 1.

### 2.2. Cytotoxicity Analysis (Extract Test)

The cytotoxicity analysis was performed in accordance with ISO 10993-5 standards, with mouse fibroblasts (L-929) and human osteosarcoma cells (MG-63) used for cytotoxicity analysis [9]. The cell culture medium employed was Dulbecco’s Modified Eagle’s Medium (DMEM; Gibco, Carlsbad, CA, USA), containing 1% antibiotics (penicillin/streptomycin/amphotericin, 100X; Gibco, Carlsbad, CA, USA) and 10% fetal bovine serum (FBS; Gibco, Carlsbad, CA, USA). The extract standard was 1.25 cm^2^/mL ± 10%, and each group of samples contained up to 3.5 mL of culture medium. The medium was placed in a 37 °C incubator, continuously shaken at 200 rpm for 72 h, and then was collected using a 0.22-μm bacteria-proof filter. The density of the cell culture was 1 × 10^5^ cells/mL/6-well plate. After being cultured for 24 h at 37 °C under 5% carbon dioxide (CO_2_), the medium was filtered and incubated for another 24 h. Subsequently, 1 mL MTT solution was added in each well. The medium was placed in the incubator for 4 h in the dark, after which 1 mL of dimethyl sulfoxide was added to each well and mixed in using a micropipette. Next, 100 μL of the liquid was drawn from the 6-well plate and deposited in the 96-well plate. Finally, an ELISA Microplate Reader (VersaMax™, Sunnyvale, CA, USA) was used to analyze and set the optical density to detect absorbance at a wavelength of 570 nm.

### 2.3. Cell Viability Analysis (Direct Contact Method)

L-929 and MG-63 cells were used for the cell viability analyses. The cell culture medium was DMEM containing 1% antibiotics and 10% FBS, and the density was 1 × 10^5^ cells/mL/6-well plate cultured on a test piece of 2 cm × 2 cm (=4 cm^2^) with a thickness greater than 1 mm. The medium was cultured for 48 h at 37 °C under 5% CO_2_, after which 1 mL MTT work solution was added to each well. The test piece was placed in the incubator for 4 h in the dark, and the MTT work solution in the six wells was then aspirated. Subsequently, 1 mL dimethyl sulfoxide was added to each well and mixed in evenly using a micropipette. Next, 100 μL of the liquid from the 6-well plate was aspirated and placed in the 96-well plate. Finally, the ELISA reader (VersaMax™) was used for analysis, with the optical density set to a 570-mm wavelength to detect the absorbance [10].

### 2.4. Alkaline Phosphatase Analysis

MG-63 cells were used for alkaline phosphatase (ALP) analysis. The cell culture medium was DMEM containing 1% antibiotics and 10% FBS, and the density was 1 × 10^5^ cells/mL/6-well plate cultured on a test piece of 2 cm × 2 cm (=4 cm^2^) with a thickness greater than 1 mm for 5 days at 37 °C under 5% CO_2_. Subsequently, 1 mL para-Nitrophenylphosphate (SouthernBiotech, Birmingham, AL, USA) was added to each well to react with the ALP and then left to rest for 30 min at room temperature. Next, 500 µL 3N sodium hydroxide was added to stop the reaction, and a micropipette was used to gently mix the solution. Then, 100 µL of the liquid was drawn from the 6-well plate and placed in the 96-well plate. Finally, the ELISA reader was used for analysis, and the optical density was set to a wavelength of 405 nm to detect the absorbance.

### 2.5. Scanning Electron Microscope

This experiment used L-929 and MG-63 for the cell viability analysis. The cell culture medium was DMEM containing 1% antibiotics and 10% FBS, and the density was 2 × 10^4^ cells/mL/6-well plate cultured on a test piece of 2 cm × 2 cm (=4 cm^2^) with a thickness greater than 1 mm. The medium was cultured for 48 h at a temperature of 37 °C under 5% CO_2_. Next, 4% neutral buffered formaldehyde (Uni-Onward, New Taipei City, Taiwan) was used to fix the solution for 15 min, after which alcohol of various concentrations was added to dehydrate the sample for 10 to 15 min. A critical point dryer (780-A, Samdri, Rockville, MD, USA) was then used to dry the sample. Finally, a coating machine (JEC-3000FC, JEOL, Tokyo, Japan) was employed for coating the sample with a layer of platinum, and a scanning electron microscope (SEM; JSM-IT 200, JEOL, Tokyo, Japan) was used to photograph the L-929 and MG-63 on six sets of test pieces at 1000× magnification.

### 2.6. RT-qPCR

In this experiment, the L-929 was used for RT-qPCR analysis. The cell culture medium was DMEM containing 1% antibiotics and 10% FBS, and the density was 5 × 10^5^ cells/mL/6-well plate. The medium was cultured on a test piece of 2 cm × 2 cm (=4 cm^2^) with a thickness greater than 1 mm for 3 days at 37 °C under 5% CO_2_. The TRIzol reagent (Invitrogen, Carlsbad, CA, USA) was used to extract and collect total ribonucleic acid, and a random primer (Invitrogen, Carlsbad, CA, USA) was applied to reverse transcribe the total ribonucleic acid into complementary deoxyribonucleic acid. SYBR Green Master Mix was mixed with the complementary deoxyribonucleic acid to perform RT-qPCR. After 10 min of pre-incubation at 95 °C, 40 cycles of 95 °C for 15 s, 40 cycles of 60 °C for 60 s, 95 °C for 15 s, 60 °C for 60 s, and 95 °C for 15 s, the comparative cycle threshold method, also known as 2^–∆∆Ct^, was employed to obtain the quantitative data on relative gene expression. The primer sequences of the Col1α1 (Collagen, type I, alpha 1), Col3α1(Collagen, type 3, alpha 1), FN1 (Fibronectin 1), and GAPDH (Glyceraldehyde-3-Phosphate Dehydrogenase) are presented in Table 1 [11,12].

### 2.7. Statistical Analysis

The statistical analysis was performed using SPSS (IBM, Armonk, NY, USA). Under the condition of a *p* value < 0.05, one-way analysis of variance was used for verification, and the differences among groups were analyzed using Scheffé’s multiple comparisons.

### 2.8. Computer Simulation Analysis

**Figure 2:**
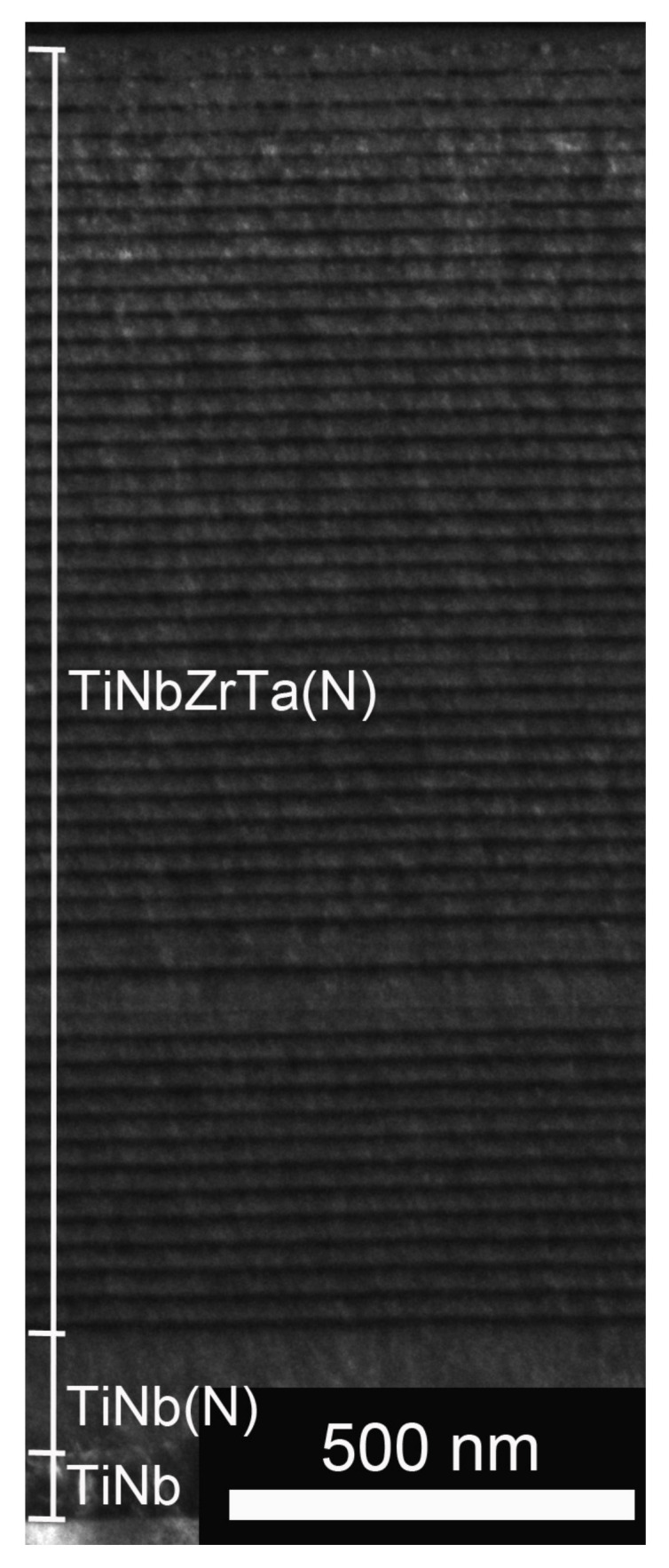
Low-magnification transmission electron microscope image of the TiNbZrTa(N) coating section.

The 3D finite element model was constructed using computer aided design software—SolidWorks 2016 (Santa Monica, CA, USA). The substrate (Ti-6Al-4V) was 20 mm in length, 20 mm in width, and 1 mm in height. However, when performing finite element analysis, the range of stress transfer did not cover the entire film. Therefore, the volume of the model was reduced to 20 μm in length, 20 μm in width, and 6 μm in height. The thickness of the coating on the films was obtained according to the dark field images of a transmission electron microscope [13], as illustrated in Figure 2. Subsequently, a computer model of a spherical indenter with a radius of 5 μm was created. The substrate Ti-6Al-4V, different coatings, and spherical indenters were combined into a new component, which formed a total of six groups, namely, Ti-6Al-4V, TiNbZr, TiNbZrTa, TiNbZrTa(N), TiNbZrTa-O, and TiNbZrTa(N)-O. Finally, they were imported into computer aided engineering software—ANSYS Workbench (Swanson Analysis, Huston, PA, USA)—for finite element analysis.

The material parameters set using finite element analysis are summarized in Table 2. All materials were set to be homogeneous and linear.

This experiment simulated the stress transfer during the nanoindentation test using nonlinear contact simulation analysis. The interface between coatings was set to a bonding state, but the spherical indenter and films were set to a contact state. The coating of the spherical indenter and top layer of the films was set to frictional (Figure 2). In addition, in reference to the data analyzed in a previous abrasion test, the frictional coefficient was set to 0.55 [12]. For interface treatment, the initial setting was for no gap between the spherical indenter and coated films, which was set to ‘adjust to touch.’

**Figure 3:**
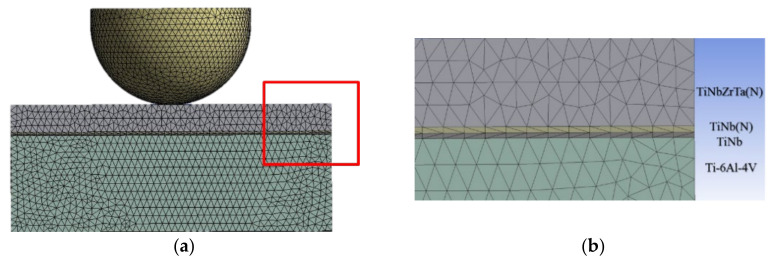
(**a**) Front view and (**b**) enlarged view of the TiNbZrTa(N) model with mesh size of 0.0004 mm.

Table 3 shows the element mesh size settings. In this study, a 10-node tetrahedral element in free mesh was used for mesh division. During convergence analysis, the maximum von Mises stress value of the TiNbZrTa series films was observed, and the error value was set to within 5%; the error value was 2.5% when the mesh was 0.0004 mm. Taking the TiNbZrTa(N) model as an example, the layers from top to bottom were TiNbZrTa(N), TiNb(N), TiNb, and Ti-6Al-4V (Figure 3).

A displacement of 200 nm was applied down the Y axis on the spherical indenter, and the X and Z axes remained unchanged. In regard to boundary conditions, the films were fixed during nanoindentation analysis, and the boundary conditions were set at the bottom of the films to lock the X, Y, and Z axes.

## 3. Results and Discussion

### 3.1. Cytotoxicity Analysis (Extract Test)

**Figure 4:**
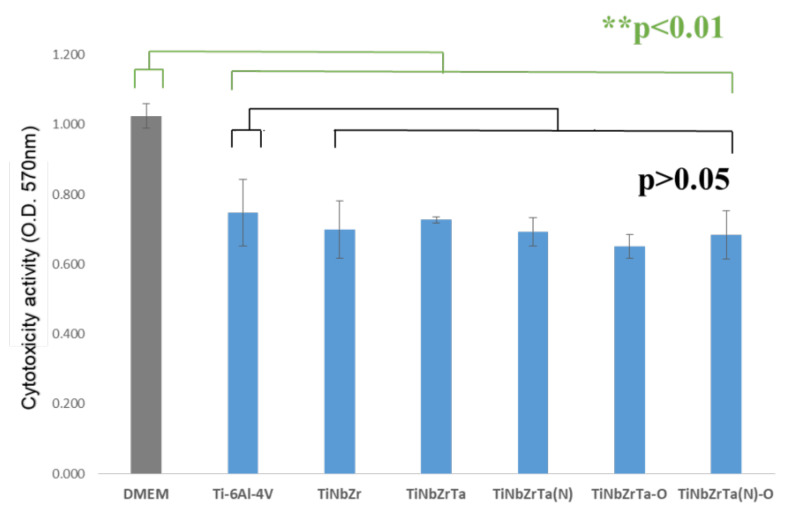
Cytotoxicity analysis of L-929 cultured on uncoated Ti-6Al-4V and TiNbZrTa series films for 48 h. DMEM acted as the medium with added serum and antibiotics without any coating extraction.

**Figure 5:**
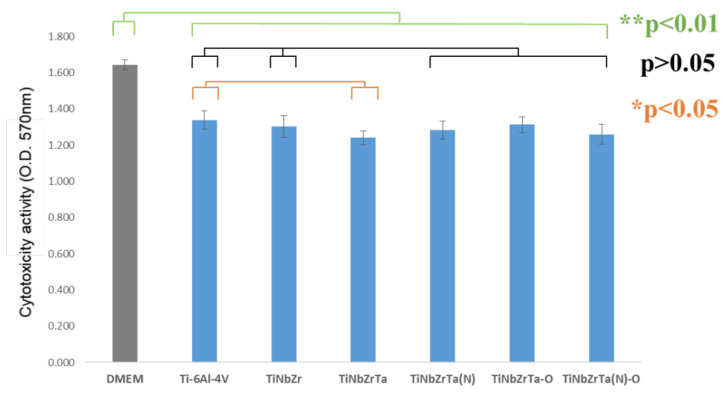
Cytotoxicity analysis of MG-63 cultured on uncoated Ti-6Al-4V and TiNbZrTa series films for 48 h. DMEM acted as the medium with added serum and antibiotics without any coating extraction.

L-929 and MG-63 were used in the cytotoxicity analyses. The MTT analysis was then performed. Higher absorbance indicated higher cell survival. According to the results, L-929 exhibited higher cytotoxicity compared with DMEM in all of the coated film media, with a significance of *p* < 0.01, as presented in Figure 4. The reason for this was that the DMEM used had added antibiotics and serum, whereas the media of the six groups of films had neither antibiotics nor serum; thus, the DMEM cells had a higher survival rate than those of the six sets of films. Because Ti-6Al-4V was recognized as a biocompatible material [5], comparing Ti-6Al-4V (uncoated) with the other coated films, no statistical significance was observed in the cytotoxicity results (*p* > 0.05), which verified that all groups of coated films and the Ti-6Al-4V were noncytotoxic. Similarly, for MG-63, the cytotoxicity of all coated film media was higher than that of DMEM, with a significance difference of *p* < 0.01, as depicted in Figure 5. Compared with Ti-6Al-4V, only TiNbZrTa was statistically significantly different (*p* < 0.05); Nb, Zr, and Ta have demonstrated to be noncytotoxic elements [17,18,19] and exhibit little difference. The results of this study revealed that all of the groups of coated films and Ti-6Al-4V were noncytotoxic.

### 3.2. Cell Viability Analyses (Direct Contact Method)

**Figure 6:**
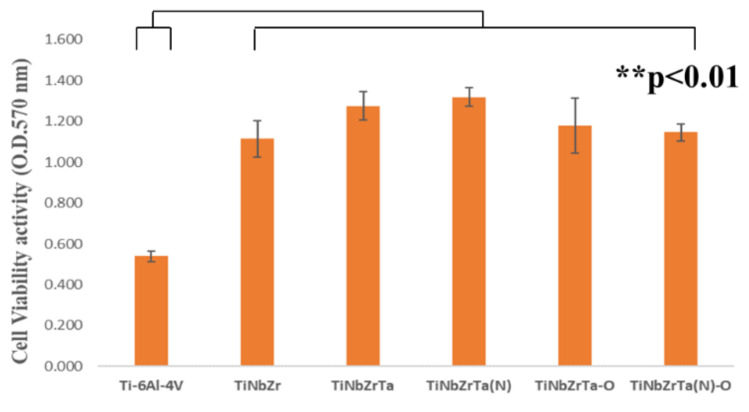
Cell viability analysis of L-929 cells cultured on uncoated Ti-6Al-4V and TiNbZrTa series films for 48 h. ** represents significance among groups (*p* < 0.01).

**Figure 7:**
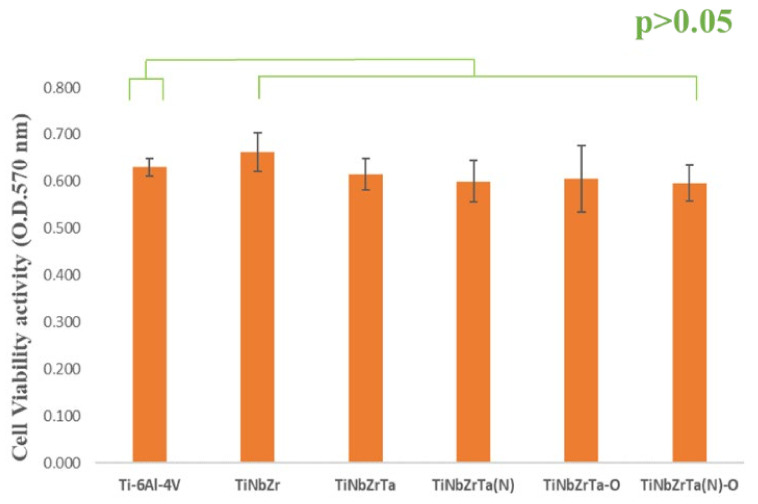
Cell viability analysis of MG-63 cells cultured on uncoated Ti-6Al-4V and TiNbZrTa series films for 48 h. No significance was noted among these groups (*p* > 0.05).

The cell viability of L-929 and MG-63 was tested to evaluate the biological activity. Both cells were cultured on the coated films for 48 h, and MTT analysis was conducted. Higher and lower absorbance indicated higher and lower cell viability, respectively. According to the results, the cell viability of L-929 cultured on all of the coated films was higher than that of the uncoated Ti-6Al-4V, with a significance of *p* < 0.01, as illustrated in Figure 6. Compared with Ti-6Al-4V, the cell viability of MG-63 cultured on all of the coated films exhibited the same trend, but was statistically nonsignificant (*p* > 0.05), as presented in Figure 7. Under the culture conditions in this study, after culturing under the same cell density and time, the proliferation rate of osteoblasts was markedly slow. These data were consistent with the previous literature [20]. The surface properties of the coatings, such as hydrophilicity and hydrophobicity or surface roughness, had an effect on cell adhesion and growth. Subsequent RT-qPCR was adopted to clarify the gene expression related to cell adhesion on the L-929-cultured coated films [17].

### 3.3. ALP Analyses

**Figure 8:**
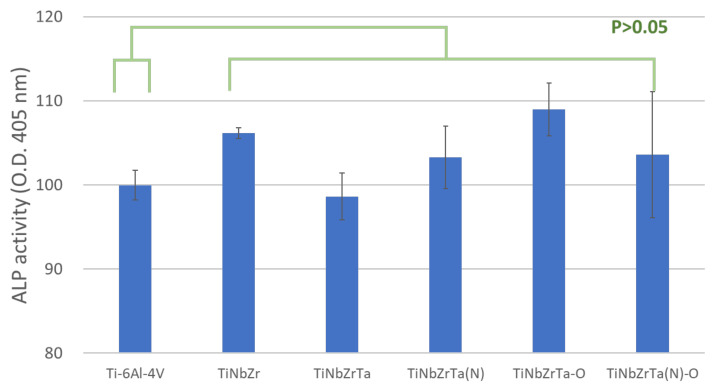
ALP analysis of MG-63 cultured on uncoated Ti-6Al-4V and TiNbZrTa series films for five days. No significant difference was noted among these groups (*p* > 0.05).

The ALP activity of MG-63 was tested to evaluate the capability of cellular differentiation. Hard tissues were cultured on the films for five days, and para-Nitrophenylphosphate was added to react with the ALP. Higher and lower absorbance indicated higher and lower ALP activity of the cell, respectively. According to the test results (Figure 8), no significance was observed among any of the groups with MG-63 cultured on the coated films or the uncoated Ti-6Al-4V (*p* > 0.05). Therefore, the results demonstrated that TiNbZrTa series films have no effect on the early osteogenic differentiation of MG-63.

### 3.4. Cell Morphology Analyses

**Figure 9:**
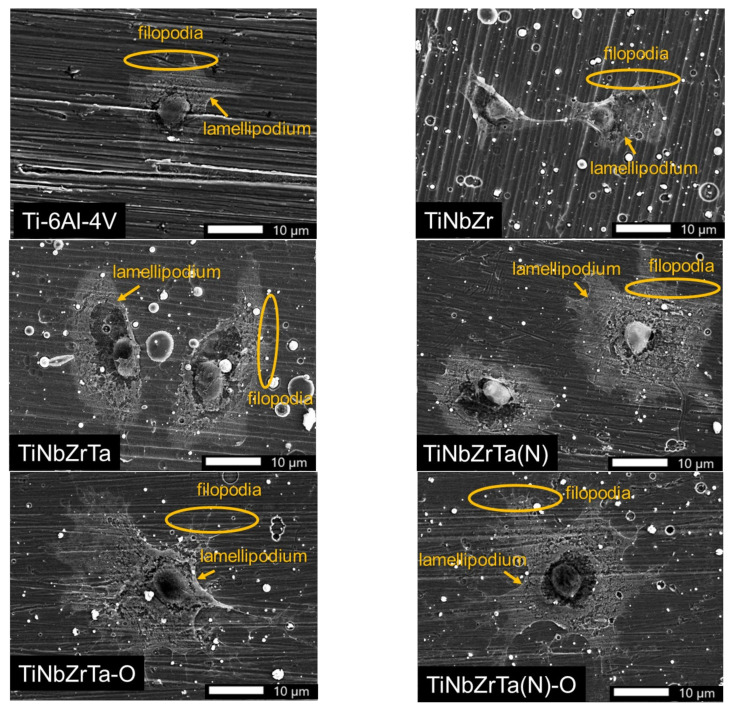
SEM images of L-929 cells cultured on Ti-6Al-4V, TiNbZr, TiNbZrTa, TiNbZrTa(N), TiNbZrTa-O, and TiNbZrTa(N)-O for 48 h. The scale was 10 µm (1000× magnification from the raw images). The arrows indicate lamellipodia, and the circles indicate filopodia.

**Figure 10:**
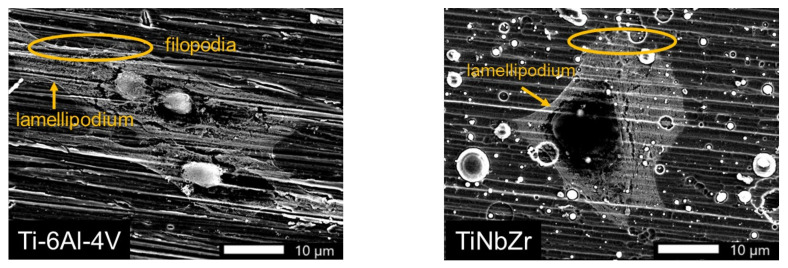

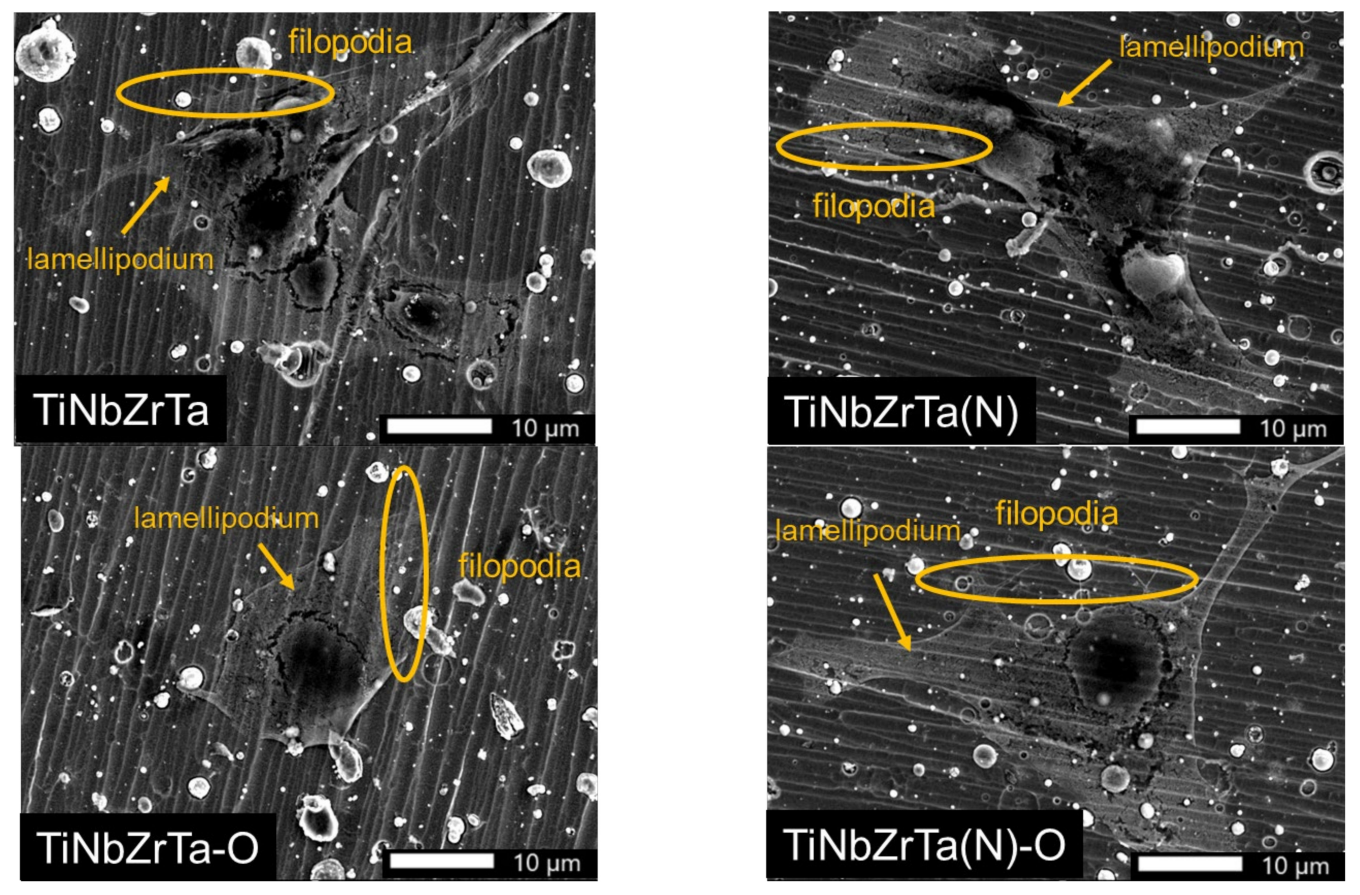
SEM images of MG-63 cells cultured on Ti-6Al-4V, TiNbZr, TiNbZrTa, TiNbZrTa(N), TiNbZrTa-O, and TiNbZrTa(N)-O for 48 h. The scale was 10 µm (1000× magnification from the raw images). The arrows indicate lamellipodia, and the circles indicate filopodia.

In this experiment, the L-929 and MG-63 were cultured on the coated films for 48 h, and the surface morphology of the cells was observed using a SEM. According to the results, whether L-929 and MG-63 were cultured on coated films or uncoated Ti-6Al-4V, the soft tissues displayed filopodia and lamellipodium (Figure 9 and Figure 10) and were firmly adhered to the six sets of films after 48 h. The cell morphology analysis results (Figure 9 and Figure 10) were consistent with the cell viability results (Figure 6 and Figure 7). L-929 cultured on the uncoated Ti-6Al-4V exhibited lower cell viability compared with that on the coated films. The SEM image revealed that the cell morphology of L-929 cultured on the uncoated Ti-6Al-4V was smaller than that of the L-929 cultured on the coated films (Figure 9). The cell viability of the MG-63 cultured on the uncoated Ti-6Al-4V was the same as that cultured on the coated films (Figure 7). The SEM image demonstrated that the cell morphologies of MG-63 cultured on the uncoated Ti-6Al-4V and coated films were of the same size (Figure 10). The actin-rich organelle protrusion at the front of the cell explored new space in the process of cell migration before adhering to the extracellular matrix or cell as part of the movement of the cell body. The most protruding structures were the lamellipodia, which are dense connective tissues formed by branched or cross-linked actin filaments [18,19]. Lamellar protuberances and folds were accompanied by the formation of parallel actin filaments, commonly called filopodia. They markedly protruded in individual cells such as fibroblasts or axon growth cones, but were also frequently observed in the free front end of the epidermis of migratory tissues [21]. The results of this study revealed that both L-929 and MG-63 easily adhered to the uncoated Ti-6Al-4V and TiNbZrTa series alloys. The coated films were more biocompatible compared with the uncoated Ti-6Al-4V because they not only facilitated cell adhesion but also maintained the normal cell diffusion structure. 

### 3.5. RT-qPCR

**Figure 11:**
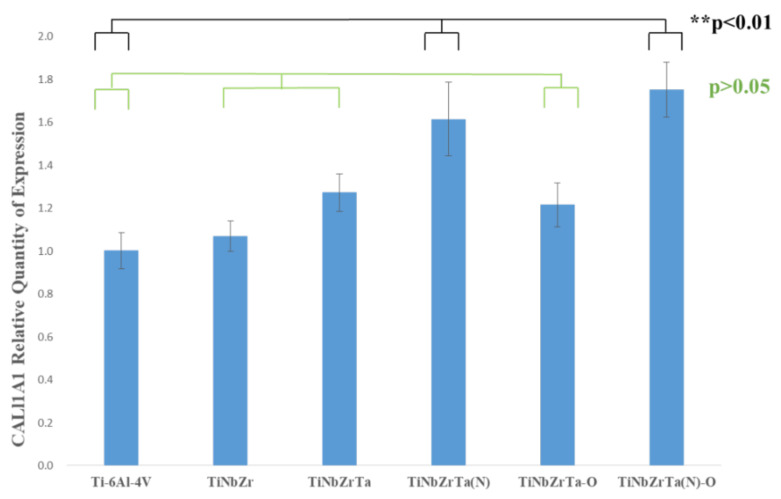
Col1α1 gene expression of L-929 cultured on uncoated Ti-6Al-4V and TiNbZrTa series films for three days. ** represents significance among groups (*p* < 0.01); no asterisk represents nonsignificance among groups (*p* > 0.05).

**Figure 12:**
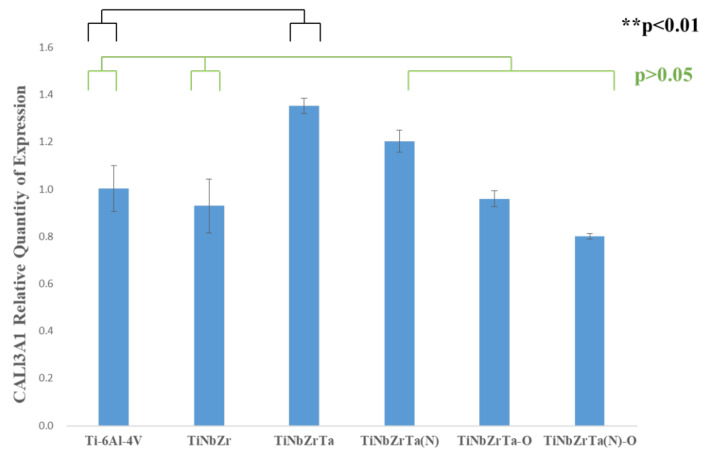
Col3α1 gene expression of L-929 cultured on uncoated Ti-6Al-4V and TiNbZrTa series films for three days. ** represents significance among groups (*p* < 0.01); no asterisk represents nonsignificance among groups (*p* > 0.05).

**Figure 13:**
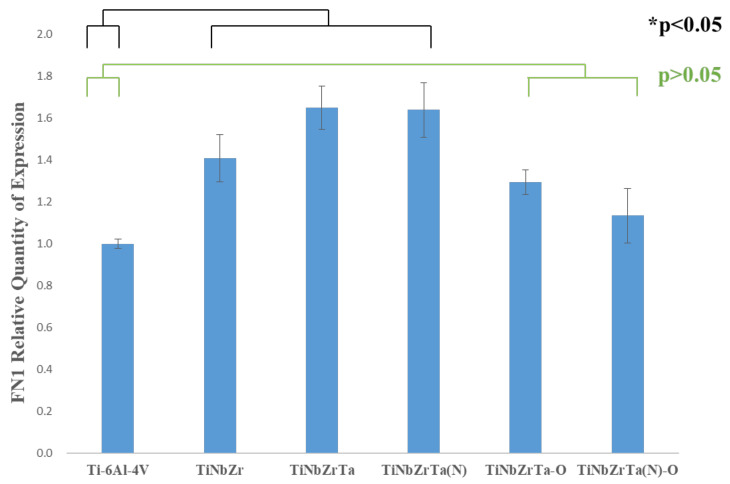
FN1 gene expression of L-929 cultured on uncoated Ti-6Al-4V and TiNbZrTa series films for three days. * represents significance among groups (*p* < 0.05); no asterisk represents nonsignificance among groups (*p* > 0.05).

In this study, L-929 was cultured on the films for three days, and the gene expressions of Col1α1, Col3α1, and FN1 were analyzed through RT-qPCR. As depicted in Figure 11, in terms of Col1α1 gene expression, the two groups, TiNbZrTa(N) and TiNbZrTa(N)-O, with nitriding coating films had a higher performance than the uncoated Ti-6Al-4V, with a significance of *p* < 0.01. However, the samples of TiNbZr, TiNbZrTa, TiNbZrTa-O, and the uncoated Ti-6Al-4V showed nonsignificance (*p* > 0.05). The Col3α1 gene expression results are presented in Figure 12. Statistically, only the TiNbZrTa coated film had a higher gene expression than the uncoated Ti-6Al-4V, and the results were significant (*p* < 0.01). Nonetheless, TiNbZr, TiNbZrTa(N), TiNbZrTa-O, and TiNbZrTa(N)-O, among the coated films, were statistically nonsignificant (*p* > 0.05). Figure 13 depicts the FN1 gene expression results. TiNbZr, TiNbZrTa, and TiNbZrTa(N) had a higher FN1 gene expression than the uncoated Ti-6Al-4V. These statistical results were significant (*p* < 0.05), whereas the TiNbZrTa-O and TiNbZrTa(N)-O coated films were nonsignificant in relation to the uncoated Ti-6Al-4V (*p* > 0.05). Col1α1 and Col3α1 were mainly produced by fibroblasts, which are the main structural proteins in the extracellular matrix of various connective tissues of animals. Collagen fibers can also guide the migration of fibroblasts during wound healing. Previous studies had reported the key role of collagen in regulating cell growth and differentiation [22,23]. The results of this study demonstrated that Col1α1 expression for L-929 cultured on the coated films increased, particularly on TiNbZrTa(N) and TiNbZrTa(N)-O. Col3α1 expression on TiNbZr, TiNbZr-O, and TiNbZrTa(N)-O was nonsignificant in relation to the uncoated Ti-6Al-4V. Only the Col3α1 of the TiNbZrTa-coated film exhibited a notable increase. These data indicated that the TiNbZrTa series alloys had a stimulating effect on collagen synthesis, particularly in the high gene expression of Col1α1. Col1α1 is the most abundant protein in different connective tissues, especially skin and bone [24]. FN1 can promote cell adhesion, repair damaged cells, and stimulate cells to secrete various functional proteins [25]. The performance of FN1 was similar to that noted in the cell viability experiments. The performance of FN1 in TiNbZrTa series films increased compared with that of FN1 on the uncoated Ti-6Al-4V. The data demonstrated that TiNbZrTa series films can improve FN1 gene expression, and FN1, in turn, can promote cell adhesion. This result was also observed in the cell morphology analysis, which verified that TiNbZrTa series alloys can enhance cell adhesion [26]. The genes regulated by these proteins were all activated, with more Col1α1 and FN1 likely released outside the cell, which was beneficial to the adhesion of L-929.

### 3.6. Microstructure Stress Analyses

**Figure 14:**
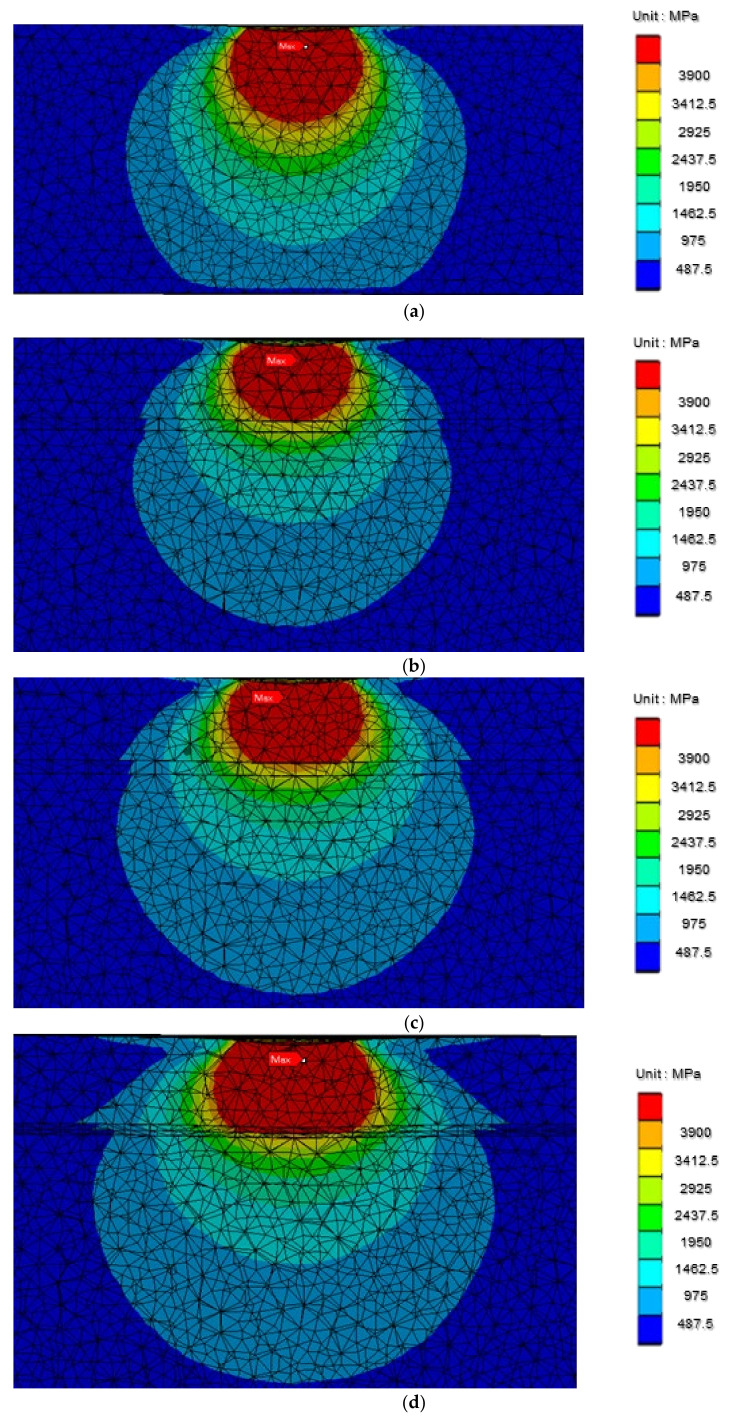

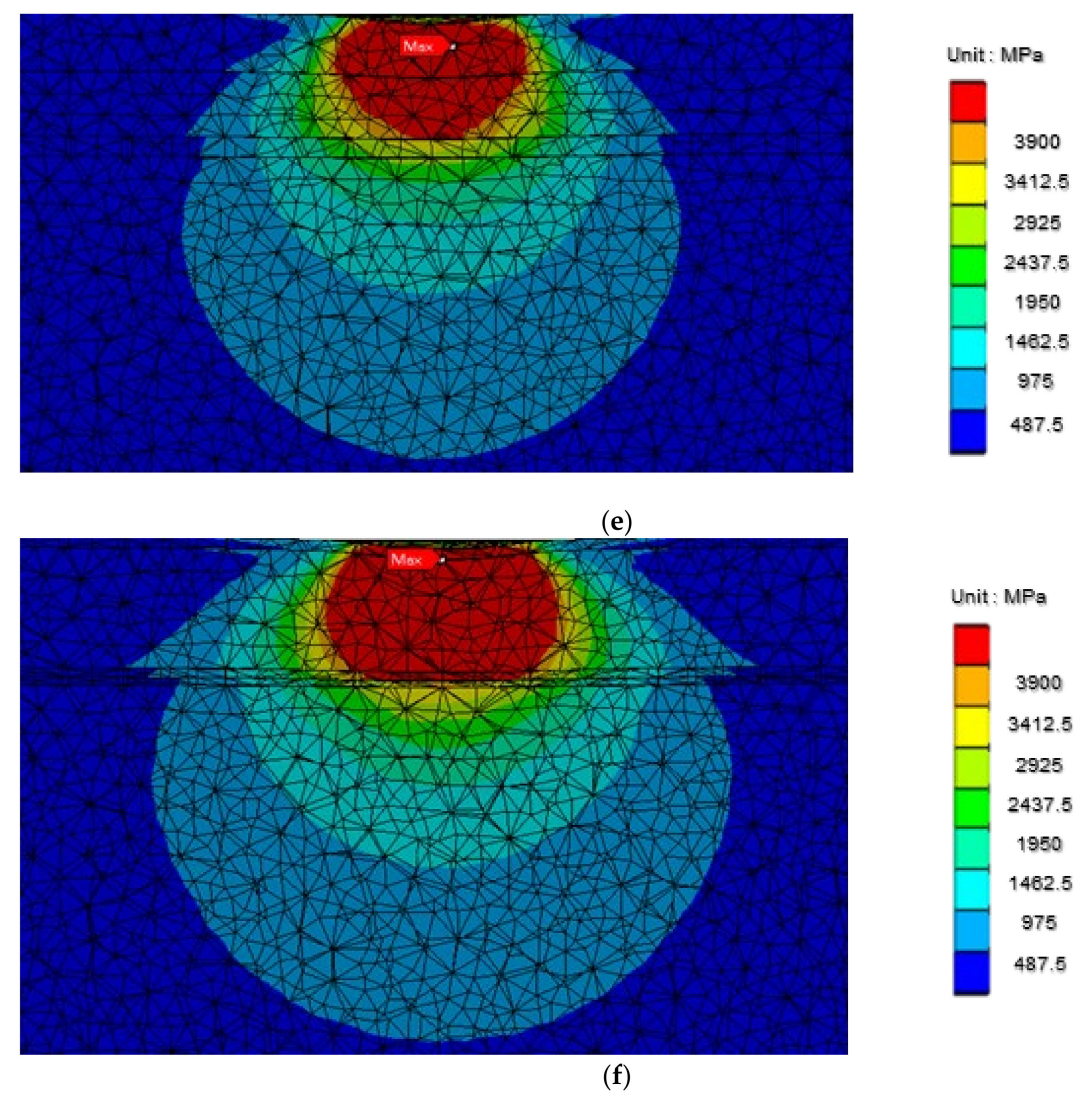
Von-Mises stress distributions of (**a**) Ti-6Al-4V, (**b**) TiNbZr, (**c**) TiNbZrTa, (**d**) TiNbZrTa(N), (**e**) TiNbZrTa-O, and (**f**) TiNbZrTa(N)-O films.

According to the results of the finite element analyses (Figure 14), the stress distribution of the uncoated Ti-6Al-4V gradually decreased from high to low stress from top to bottom. For the TiNbZr coated films, high stress was present in the uppermost TiNbZr coating. The stress, when transferred to the TiNb coating, was considerably reduced. Similarly, the stress distribution of the TiNbZrTa coated film manifested as high stress in the uppermost TiNbZrTa coating and was considerably reduced when the stress was transferred to the TiNb coating. For the TiNbZrTa(N) coated film, high stress was present in the uppermost TiNbZrTa(N) coating and underlying TiNb(N) coating. The stress was notably reduced when it transferred to the TiNb layer. Regarding the TiNbZrTa-O-coated film, high stress was also observed in the uppermost TiNbZrTa-O coating and underlying TiNbZrTa. When transferred to the TiNb layer, the stress reduced considerably. For the TiNbZrTa(N)-O-coated film, high stress manifested in the uppermost TiNbZrTa(N)-O coating, underlying TiNbZrTa(N) layer, and TiNb(N) interlayer. The stress was markedly reduced when it transferred to the TiNb bottom layer.

According to the results (Figure 14), stress distribution in the uncoated Ti-6Al-4V gradually changed from high to low stress (Figure 14a). However, in this study, the five sets of coated films (Figure 14b–f) exhibited obvious stress reduction when the stress was transferred from the upper coating to the lower TiNb layer because of the low Young’s modulus of TiNb. As illustrated in Figure 14d–f (coated films with nitrogen doping), the Young’s modulus of the TiNb(N), TiNbZrTa(N), and TiNbZrTa(N)-O coatings was higher than that of other coatings; however, the experimental results did not affect the stress transfer phenomenon. This result was consistent with the study of Zhao et al. [27], where the introduction of a multilayer structure changed the stress field. In this study, the intermediate layer of TiNb in the five groups of films reduced the stress concentration of the internal structure of the overall coating and may have reduced the risk of coating deformation and fracture resulting from excessive stress [27,28].

### 3.7. Research Limitations

The cell viability and ALP analyses led to the selection of MG-63, with its slow growth, for further experimentation. In relevant studies, experiments were performed on osteoblasts for at least seven days to observe the changes [29,30]. Because the duration of this study was short, the potential to observe the difference in trends between the experimental and control groups was limited. In the finite element analysis, coating thickness of the film was obtained using a field emission transmission electron microscope. Through this analysis, the thickness and finer layer thickness of the top layer of each group of the coated films in the field emission transmission electron microscope images could be identified. However, because the material parameters and properties of each group of the finer top layers could not be obtained, analyses of the stress distribution within the coating could not be conducted comprehensively.

## 4. Conclusions

The ISO-10993-5 cytotoxicity test verified that the TiNbZrTa series films had the same results for L-929 and MG-63 and on the uncoated Ti-6Al-4V, indicating that the TiNbZrTa series films were noncytotoxic. MTT cell viability analysis on L-929 and MG-63 demonstrated strong biocompatibility, particularly for L-929. In the ALP analysis, the results of MG-63 cultured on the uncoated Ti-6Al-4V and TiNbZrTa series films were similar, indicating that the TiNbZrTa series films had no effect on the early osteogenic differentiation of MG-63. Cell morphology analyses revealed that both L-929 and MG-63 presented filopodia and lamellipodium. This signified that both soft and hard tissues could easily adhere to the uncoated Ti-6Al-4V and TiNbZrTa series films. RT-qPCR verified that L-929 which was cultured on the TiNbZrTa series films exhibited high Col1α1 and FN1 gene expression, indicating that the regulation of cell growth, differentiation, and adhesion was improved. The results of the finite element analysis showed that, compared with the uncoated Ti-6Al-4V, the TiNbZrTa series films could reduce the stress concentration inside the coating, with the five groups of coated films exhibiting the same effect induced by the intermediate TiNb coating with a low Young’s modulus. Therefore, the stress distribution of different coatings can be assessed in advance. The results of this study revealed that TiNbZrTa series films provided a satisfactory microenvironment for the survival and growth of cells. In addition, when the coating was subjected to load, the accumulation of excessive stress within the coatings might be prevented. The TiNbZrTa series films are expected to be developed and applied to biomedical materials in the future.

## Figures and Tables

**Table 1:** qPCR primer sequence and product size.

Gene	Sequence of Primers (5’-3’)	bps
*Col1α1*	F-5’-ACTGTCTTGCCCCAAGTTCC-3’	124
	R-5’-TGGGCATCTGGTTTAGCCTT-3’	
*Col3α1*	F-5’-ACGTAGATGAATTGGGATGCAG-3’	154
	R-5’-GGGTTGGGGCAGTCTAGTG-3’	
*Fn1*	F-5’-CCGGGTGTCCTGATCGTT-3’	120
	R-5’-GGGGAGACCTGGGAAAAG-3’	
*GAPDH*	F-5’-TATGTCGTGGAGTCTACTGGT-3’	129
	R’-5-GAGTTGTCATATTTCTCGTGG-3’	

**Table 2:** Material parameters of coating and indenter [13,14,15,16].

Material	Young’s Modulus (GPa)	Poisson’s Ratio
Ti-6Al-4V	144.4	0.34
TiNb	120.2	0.36
TiNb(N)	206.6	0.36
TiNbZr	111.1	0.34
TiNbZrTa	130.8	0.36
TiNbZrTa(N)	180.6	0.36
TiNbZrTa(N)	124.5	0.36
TiNbZrTa(N)-O	176.4	0.36
Inderter	1140	0.07

**Table 3:** Mesh size setting (mm).

Ti-6Al-4V	0.0004
Film	0.0004
Indenter	0.0004

## Data Availability

The data that support the findings of this study are available from the corresponding author upon reasonable request.
